# Enabling thin-film transistor technologies and the device metrics that matter

**DOI:** 10.1038/s41467-018-07424-2

**Published:** 2018-12-10

**Authors:** Alexandra F. Paterson, Thomas D. Anthopoulos

**Affiliations:** King Abdullah University of Science and Technology (KAUST), Division of Physical Sciences and Engineering and KAUST Solar Centre, Thuwal, 23955-6900 Saudi Arabia

## Abstract

The field-effect transistor kickstarted the digital revolution that propelled our society into the information age. One member of the now large family of field-effect devices is the thin-film transistor (TFT), best known for its enabling role in modern flat-panel displays. TFTs can be used in all sorts of innovative applications because of the broad variety of materials they can be made from, which give them diverse electrical and mechanical characteristics. To successfully utilize TFT technologies in a variety of rapidly emerging applications, such as flexible, stretchable and transparent large-area microelectronics, there are a number of metrics that matter.

## Introduction

**Fig. 1:**
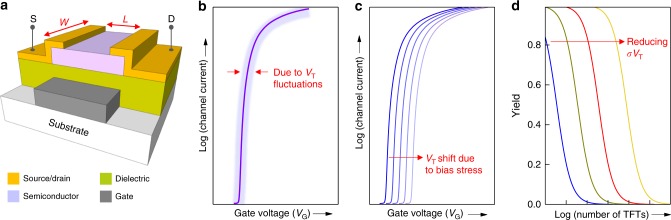
Thin-film transistor architecture and the important device metrics. **a** Generic schematic of a staggered bottom-gate TFT. **b** Conceptual transfer curve (solid line) and characteristic spread (shaded region) attributed to threshold voltage fluctuations for a large set of TFTs. **c** Conceptual illustration of the transfer curve shift due to continuous bias application. **d** Approximate plot of the circuit yield as a function of the number of concatenated unipolar NOT gates using *σV*_T_ as the only variable parameter. The plot shows that the less *V*_T_ fluctuates (i.e. *σV*_T_ becomes smaller), the larger the number of logic gates/TFTs that can be integrated onto the same circuit

Looking around us one thing is clear; optical displays have become an integral part of defining and shaping our modern living environment. The role of displays in gradually transforming our everyday landscape began in the 1980’s, when liquid crystal-based displays became popular, and the thin-film transistor (TFT) (Fig. 1a) finally found its calling^1^. The TFT was the device of choice for driving the individual picture elements (pixels) then and remains so now, with TFT driven backplanes being at the heart of the display industry. Today, and after several decades of research and development, the amorphous silicon (a-Si) TFT remains the primary technology used to drive liquid crystal displays (LCDs). However, as new display technologies are being developed, the focus of TFT research is shifting to new semiconductor materials—a move spurred on by shortcomings of the established technologies, or the beneficial properties offered by new families of materials, like metal oxides and organic semiconductors^2,3^.

## Charge carrier mobility

Regardless of which semiconductor is chosen for TFTs, there is one metric that is often considered to be the most important: the charge carrier mobility (*µ*). The mobility characterises how swiftly charge carriers can move through a given semiconductor and, although considered an intrinsic property, TFT-measured mobility is known to depend on various extrinsic factors. Mobility values vary wildly between materials families (Table 1) and in general, there is a typical TFT ‘mobility ethos’ of the bigger the number, the better the transistor, because the higher the carrier mobility, the more applications the TFT can be used for. This has not been good news for carbon-based organic semiconductors, which, for a long time, had mobility values that were a world away from their inorganic counterparts. But the same peculiar chemical nature that is responsible for their low mobilities also gives organic semiconductors extreme processing versatility, as well as unique mechanical properties, such as flexibility and stretchability. The latter qualities spawned the idea of a foldable, flexible electronics market that captured the imagination of people throughout the world. This enthusiasm for flexible, printed electronics has driven huge research efforts to improve the critically low mobilities of organic semiconductors, measured in organic TFTs (OTFTs).

**Table 1:** Performance and characteristics of TFTs made from different semiconductors

TFT properties	Semiconductor			
	Organic	α-Si	Poly-Si	Metal oxides
Mobility (cm^2^/Vs)	1–20	0.1–1	Up to 100	Up to 100
Lifetime stability	Low	Low	High	High
Uniformity	Low	High	Low	High
Commercial applications	–	LCD displays	Smart phone displays	Laptop displays
Costs	Cheap	Cheap	Expensive	Cheap
Potential for large area application	Excellent	Poor	Poor	Excellent

Nevertheless, it is the high charge carrier mobility values for OTFTs that have been in the spotlight in recent years — for all the wrong reasons. In a haze of glory, excitement and misinterpretation, overestimated OTFT mobility values gradually started creeping into the literature circa 2004, until they were being published frequently at the peak of the mobility hype (2012–2017)^2^. The quest for higher mobility numbers has been driven by the desire to use OTFTs in as many applications as possible, including emerging foldable, stretchable and flexible opto/electronics. However, understanding the fundamental nature of charge transport in the myriads of organic semiconductors has, in many ways, proven to be a challenge—particularly when it comes to field-effect carrier mobility interpretation. With the mobility hype only recently going from hot potato to hot topic, all are in agreement that proper mobility analysis from considered data is critical. And despite this brief interruption, OTFT mobility values have made remarkable improvements, with reliable values going from 0.00001 to over 20 cm^2^/Vs in the past 30 years^3^. But how much does mobility matter? Has mobility been hogging the limelight for so long that is has detracted attention from other important metrics?

## Channel miniaturisation

The ultimate goal at the end of the TFT research journey is their successful deployment in optical displays and integrated circuits (ICs) for a broad range of emerging applications. For the former, brightness, glare and colour blending can be improved in high definition (HD) displays by improving the so-called ‘fill factor’, or reducing the size of the black area of a pixel, where the black area is the non-emissive part of the pixel that contains the driving circuitry (capacitor & TFTs), the pixel interconnects and interdot spacing^4^. One approach for increasing the pixel fill factor is to reduce the size of the TFTs. This is done either by shortening the channel length (*L*) and/or increasing the mobility of the semiconductor, which in turn allows reduction of the channel width (*W*) (Fig. 1a) without reducing the current-driving capabilities of the device. Reducing *L* and improving *µ* can also be used to increase the operating frequency of ICs, since the maximum switching speed (*f*_C_) of the individual TFTs is related to both *L* and *µ* via $f_{\mathrm{C}} \propto \frac{{\mu \times V_{\mathrm{D}}}}{{L^2}}$, where *V*_D_ is the applied source-drain potential^5,6^. Channel miniaturization has therefore long been a popular topic for all types of transistor technologies, and formed the basis of Moore’s famous law.

In the context of channel miniaturization, the general transistor mobility ethos of the bigger the number, the better the device, starts to become untrue. In long-channel TFTs (>5 µm), charge carrier mobility is a good metric for gauging the suitability of a given material for a specific application, because *L* is long enough for the semiconductor to dominate charge transport across the device. However, as *L* reduces, transistor operation starts to deviate from its ideal behaviour and becomes dominated by potential barriers present at the injecting contacts and the associated contact resistances, *R*_C_. In situations where *R*_C_ exceeds that of the gate-field dependent channel resistance, *R*_Ch_(*V*_G_), analysis of the carrier transport using the standard transistor model^7^ is no longer applicable, and the measured mobility is often considerably lower than in long-channel devices^6,8,9^. The impact of injection limited operation is known to be particularly pronounced in TFT families where intentional doping of the contacts is technologically challenging, with organic transistors being a representative example^2^.

It turns out that for many emerging TFT technologies, parasitic contact resistance effects tend to dictate device operation, resulting in *μ* often being significantly lower than the semiconductor’s intrinsic value. Consequently, for the majority of envisioned TFT-based applications, *L* and related *R*_C_ are more critical metrics than *µ*. Indeed, this resonates with the story behind the metal-oxide-semiconductor field-effect transistor (MOSFET), for which it was initially believed that higher *μ* was the answer for achieving better performing ICs^8^. However, it turned out that channel miniaturization—not higher *µ*—gave the desired improvements in device speed and transistor integration density^10^.

## Transistor parameter variation

Having said that, it will not matter how small *L* and associated *R*_C_ are, if the operating parameters fluctuate between individual TFTs. Importantly, for any ICs, if TFT parameter variability is too great then it won’t be possible to integrate thousands or even millions of devices onto one functional circuit. In digital circuits, the gate voltage applied to make the TFT channel conduct electricity (the so-called threshold voltage, *V*_T_) is critical, with its variability between individual devices, i.e. standard deviation, *σV*_T_, being the most important metric out of all other influential parameters^10,11^. Figure 1b, c illustrates how the transfer curves for a large set of identical TFTs may deviate (shaded area) from the ideal conceptual characteristic (solid line) due to *V*_T_ fluctuations. The origin of *V*_T_-variation can, in most cases, be traced to either manufacturing process (Fig. 1b) or the operational stability of the device (Fig. 1c), and in the case of flexible devices and circuits, can arise from mechanical bending. If one excludes the latter, then the degree of *V*_*T*_ fluctuation depends heavily on the semiconductor used. For example, high mobility poly-Si TFTs suffer from manufacturing associated *σV*_T_ (Fig. 1b), but they are well-known for their excellent operational stability. On the other hand, a-Si TFTs are infamous for their operational instability (Fig. 1c) and low carrier mobility, but renowned for their high parameter uniformity (small *σV*_T_) even when manufactured over large area substrates (Table 1).

In TFT backplanes for displays, *V*_T_ variations affect the brightness of the individual pixels and cause luminance variation across the display. In ICs, a large *σV*_T_ not only causes delays in circuitry^12^, but in some cases, such as unipolar ICs, the magnitude of *σV*_T_ actually dictates the maximum number of TFTs that can be integrated onto a circuit. Therefore, the more complex an IC becomes, the more important *σV*_T_ gets^13^. Figure 1d illustrates this by showing the impact of *σV*_T_ as a single parameter on circuit yield, as a function of the number of concatenated unipolar logic NOT gates. To overcome this rather serious integration bottleneck, pseudo-complementary or complementary circuitry are often employed, but unfortunately at the expense of manufacturing complexity and cost^14^. Similarly, in displays, *V*_T_ shift in a-Si TFT-based pixels is managed with additional transistors in the pixel circuit, although this adversely affects the fill-factor^4,10,15^. On the whole, considering multiple TFT parameters together—not only *µ*—builds a picture of how suitable the various emerging TFT technologies are for the different applications.

## Transistor metrics that matter

It is evident that channel miniaturisation and large-scale integration are important for the future of TFTs. Achieving the highest possible carrier mobility is often considered an important goal for this particular transistor technology. However, if TFTs are put into real-life context of optical displays and other emerging electronic products, then channel length, contact resistance and threshold voltage fluctuations become equally important—if not more important—metrics than carrier mobility. For OTFTs in particular, so much focus has been placed on achieving the highest *µ* that it may have detracted attention from other important metrics, such as operating instability, large *R*_C_ and manufacturing uniformity. Although *µ* and *V*_T_ are reported as standard in the vast majority of the relevant TFT literature (for organic and inorganic semiconductors), high mobility values that are reported out of context run the risk of becoming abstract numbers. If emerging TFT technologies are to make it into commercial applications, then putting performance metrics into context becomes critical.

## References

[1] Brody TP (1984). The thin film transistor: a late flowering bloom. IEEE Trans. Electron Devices.

[2] Paterson Alexandra F., Singh Saumya, Fallon Kealan J., Hodsden Thomas, Han Yang, Schroeder Bob C., Bronstein Hugo, Heeney Martin, McCulloch Iain, Anthopoulos Thomas D. (2018). Recent Progress in High-Mobility Organic Transistors: A Reality Check. Advanced Materials.

[3] Labram JG, Lin YH, Anthopoulos TD (2015). Exploring two-dimensional transport phenomena in metal oxide heterointerfaces for next-generation, high-performance, thin-film transistor technologies. Small.

[4] Kumar A, Nathan A, Jabbour GE (2005). Does TFT mobility impact pixel size in AMOLED backplanes?. IEEE Trans. Electron Devices.

[5] Wang JZ, Zheng ZH, Sirringhaus H (2006). Suppression of short-channel effects in organic thin-film transistors. Appl. Phys. Lett..

[6] Klauk, H. Will we see gigahertz organic transistors? *Adv. Electron. Mater*. **4**, 1700474 (2018).

[7] Shockley W. (1952). A Unipolar "Field-Effect" Transistor. Proceedings of the IRE.

[8] Franklin AD (2015). Nanomaterials in transistors: from high-performance to thin-film applications. Science.

[9] Marinkovic M, Belaineh D, Wagner V, Knipp D (2012). On the origin of contact resistances of organic thin film transistors. Adv. Mater..

[10] Street RA (2009). Thin-film transistors. Adv. Mater..

[11] Liu, H., Xie, J., Tian, M. & Deng, A.-C. Fast monte carlo statistical analysis using threshold voltage modeling. US patent US13939117 (2015).

[12] Wirnshofer Martin (2013). Sources of Variation. Variation-Aware Adaptive Voltage Scaling for Digital CMOS Circuits.

[13] Terada, K. & Mogami, T. Measurement of Standard Deviation for Threshold Voltage using Parallel-Connected MOSFETs. In *ESSDERC '96: Proc. of the 26th European Solid State Device Research Conference*, 499–502 (IEEE, 1996).

[14] Gelinck G, Heremans P, Nomoto K, Anthopoulos TD (2010). Organic transistors in optical displays and microelectronic applications. Adv. Mater..

[15] Kuo Y (2013). Thin Film Transistor Technology—Past, Present, and Future. Electrochem. Soc. Interface.

